# Nurses’ perception of the multidisciplinary team approach of care for adolescent mothers and their children in Ugu, KwaZulu-Natal

**DOI:** 10.4102/phcfm.v11i1.1936

**Published:** 2019-04-23

**Authors:** Desiree Govender, Saloshni Naidoo, Myra Taylor

**Affiliations:** 1 KwaZulu-Natal Department of Health, Ugu District, South Africa; 2 School of Nursing and Public Health, Discipline of Public Health Medicine, University of KwaZulu-Natal, Durban, South Africa

**Keywords:** adolescent pregnancy, multidisciplinary care, healthcare providers, teamwork, knowledge exchange

## Abstract

**Background:**

Adolescent childbearing has numerous consequences on maternal health, child health and the well-being of society. Because of the high-risk nature of adolescent pregnancy, a multidisciplinary team (MDT) approach is recommended to achieve satisfactory pregnancy outcomes.

**Aim:**

The aim of this study was to explore nurses’ perceptions of the MDT approach in the continuum of care for adolescent mothers and their children.

**Setting:**

The study was conducted in a local district hospital in Ugu, KwaZulu-Natal.

**Methods:**

An explorative and descriptive qualitative study design was used. The first author and hospital staff (clinical midwives and the clinical manager of obstetrics and gynaecology) collaborated on the development of the focus group discussion (FGD) guide to explore nurses’ perception of the MDT approach of care for adolescent mothers and their children. A total of three FGDs were conducted. Data were analysed using thematic analysis.

**Results:**

Six overarching themes emerged from the data analysis which included professional benefits of adopting the MDT approach of care for adolescent mothers and their children, barriers to the multidisciplinary collaboration, clinical benefits of adopting the MDT approach of care for parenting adolescent mothers, problems and needs of adolescent mothers, and nurses’ reasons regarding their willingness to participate in a multidisciplinary collaboration in the care of parenting adolescent mothers.

**Conclusion:**

In the opinion of nurses, the MDT approach of care for adolescent mothers and their children is an important strategy to improve maternal and child health outcomes. This study has important implications for the design of an intervention.

## Introduction

Adolescent pregnancy is a complex and multifaceted public health issue.^1^ Early childbearing poses a challenge to the eradication of poverty, universal primary education, gender equality and women’s empowerment, reduction of child mortality, improvements in maternal health, and the struggle with the human immunodeficiency virus (HIV) and acquired immune deficiency syndrome (AIDS).^2,3^

A retrospective cohort study carried out in Manitoba, Canada, identified that children of adolescent mothers had a higher risk of hospitalisation, high hospital use, academic failure and poor social skills.^4^ Furthermore, evidence concluded that the children of the adolescent mothers accounted for 27% of hospitalisation cases in the first year, while 34% had experienced early mortality (birth to 17 years). The study further emphasised the vulnerability of adolescent parents and their children to social problems, with 51% of children in foster care, while 44% were dependent on welfare and 36% became adolescent mothers. It is therefore evident that adolescent motherhood drives the social problems from one generation to another.

The children of adolescent mothers are also prone to neglect, accidental injuries and speech and language delays. The research conducted by Keown et al.^5^ in New Zealand found that maternal intrusiveness and maternal involvement with the child accounts for the relationship between adolescent motherhood and poor language development. Maternal intrusiveness is defined as ‘frequent, non-contingent physical behaviour or verbal directives that limit the child’s activities’ (p. 195).^6^ Therefore, speech language screening is recommended on a regular basis so that early intervention can be implemented.

Thompson^7^ asserts that an adolescent mother and her child are considered as two individual paediatric patients, with each requiring specialised health care. Because of the high-risk nature of adolescent pregnancy, the multidisciplinary team (MDT) approach is recommended to achieve satisfactory pregnancy outcomes.^8^ Multidisciplinary care is defined as ‘care that occurs when professionals from a range of disciplines work together to deliver comprehensive care that addresses as many of the patient’s health and other needs as possible’ (p. S61).^9^

Pinzon and Jones^10^ have also emphasised the usefulness of multidisciplinary collaboration in the care of parenting adolescent mothers and their children. Quinlivan and Evans^11^ conducted a study in three Australian hospitals that investigated the impact of a multidisciplinary antenatal clinic for pregnant adolescents on birth outcomes, breastfeeding postpartum and contraception uptake. The various healthcare providers at the multidisciplinary antenatal clinic included psychologists, social workers, dieticians, psychiatrists, doctors and clinical midwives. Adolescent mothers (attendees of the multidisciplinary clinic) in the study had a significantly lower risk of threatened premature labour (OR: 0.45; 95% CI: 0.29–0.68, *p* < 0.0001), premature rupture of membranes (OR 0.34; 95% CI 0.18–0.6, *p* = 0.0002) or premature delivery (OR: 0.40; 95% CI: 0.25–0.62, *p* < 0.0001) compared with adolescent mothers from general clinics.^11^ Postpartum uptake of contraception was higher among the adolescent mothers attending the clinic (OR: 1.58; 95% CI: 1.07–2.25, *p* < 0.01).

South African adolescent females are not immune to the negative consequences of early childbearing. Pregnant adolescents attending a district hospital in northern KwaZulu-Natal underutilised antenatal services (< 4 visits).^12^ The underutilisation of antenatal services by pregnant adolescents was significantly associated with lower gestational age (< 37 weeks) (OR: 2.64; 95% CI: 1.04–6.74, *p* < 0.05). Adolescent mothers in this study also reported emotional vulnerability and the burden of stigma related to adolescent pregnancy in society.^12^ Similarly, Sodi and Sod,^13^ gathered from their study, conducted in Limpopo, South Africa, that adolescent mothers experience elevated emotional problems, negative social relationships, disruption in schooling and intense lifestyle changes. Drawing from the South African context, nurses are the first-line of practitioners who have been tasked with the provision of sexual and reproductive health services to adolescents, but multifaceted problems, such as adolescent pregnancy, are not limited to nursing care.

According to Thompson^7^, the basic needs of the adolescent mother and her child include growth and development screening, immunisation, nutrition screening, acute illness management, lactation support, psychosocial assessment, provision of birth control, sexual health education, infant mental health screening, early childhood interventions, child safety and behaviour support, parenting support and maternal mental health screening.

Understanding the perceptions of the multidisciplinary care approach for adolescent mothers and their children in the South African context is a vital step in supporting clinical practice. This study explored nurses’ perceptions of the MDT approach of care for adolescent mothers and their children.

## Research methods

### Study design

This research was part of the first phase of a larger PhD study titled ‘A community of practice model for a multidisciplinary and comprehensive approach towards caring for the parenting adolescent mother’. The larger PhD study encompassed a mixed-methods action research study (MMAR). For the purpose of this research, a descriptive qualitative study was used to explore nurses’ opinions and perceptions of the MDT approach of care to possibly introduce an intervention. The descriptive qualitative study also prepared for the action research process as an environmental scan. It is widely acknowledged that action research is a method that can improve practice and the working environment.^14^ Reason and Bradbury^15^ define action research as ‘a participatory process with developing practical knowing in the pursuit of worthwhile human purposes’ (p. 8). In the healthcare setting, action research has the ability to positively impact healthcare clinical practice and health outcomes for users. Focus group discussions (FGDs) were conducted for data collection.

### Context of the study

The principal researcher of this study (D.G.) has worked as a physiotherapist for 10 years at a 300 bedded local district hospital in Ugu, KwaZulu-Natal. The services offered by the hospital include obstetrics and gynaecology, surgery, orthopaedics, paediatrics, mental health, dietetics, rehabilitation, radiology and social work services. The hospital provides healthcare services to the residents of Umdoni. Umzumbe and Vulamehlo. The principal researcher had the opportunity of interacting with adolescent mothers while rendering physiotherapeutic services to them in the postnatal ward and to their infants in the nursery. She has also observed the increase in the adolescent delivery rate (females 19 years and younger) at the hospital. The adolescent delivery rate in Ugu district is currently 23%. Adolescent females attending the antenatal clinic and labour ward do not always present for a first pregnancy but for repeat pregnancies.^16^

The principal researcher’s engagement with adolescent mothers and her growing awareness of the multitude of problems (medical, psychological, socio-economic) experienced by adolescent mothers and their children motivated her to probe into the nature of their current care at her institution. After informal discussions with the medical doctors, clinical midwives, nursing managers, a clinical psychologist and social workers, it was apparent that there were no specific services in the hospital tailored for adolescent pregnancy, adolescent mothers or their children. Nurses were the first-line of contact for pregnant and parenting adolescents.

### Study population

The population of interest were all professional nurses, enrolled nurses and auxiliary nurses caring for the health of pregnant adolescents, adolescent mothers and their children, in the health facility. Three wards that were identified included the antenatal clinic, the postnatal ward and the nursery with a total of 24 nursing staff.

### Sampling method

The antenatal, the postnatal and nursery units were purposefully identified because the research interest was the care of pregnant adolescents, parenting adolescents and their children. The principal researcher contacted all operational managers of the antenatal, postnatal and nursery units to obtain permission to inform staff about the research study. This study included a sample of volunteer nurses from these identified units to participate in the study. Therefore, all professional nurses, enrolled nurses and auxiliary nurses who were willing to voluntarily participate in this study were included in the FGDs.

### Sample size

A total of three FGDs were conducted. Focus group 1 consisted of eight participants, while focus groups 2 and 3 consisted of six participants each, making a total of 20 participants. The number of participants was limited and therefore the focus groups were few. However, data saturation was reached by the third focus group.

### Data collection

To ensure the trustworthiness of the data, the focus group discussion guide (FGDG) was developed with the clinical manager (obstetrics and gynaecology) and three clinical midwives during research meetings (see Box 1). The FGDG was also checked by the research supervisors. Prior to data collection, the principal researcher became familiar with the units within the organisation who participated in the FGDs. Initially, the research idea was introduced to nursing staff during ward meetings to give them the opportunity to decide if they wanted to participate in the FGDs. This ensured that individuals who participated in this study were sincerely interested in sharing their perspectives.

BOX 1 Focus group discussion guide.

What do you think are the professional benefits of adopting the multidisciplinary team care approach for adolescent mothers and their children?

What do you consider to be the potential barriers to multidisciplinary collaboration?

What do you think are the clinical benefits of adopting the multidisciplinary team care approach for adolescent mothers and their children?

What are the problems and needs of parenting adolescent mothers?

Why would you consider participating in a multidisciplinary collaboration of care for adolescent mothers and their children?

With regard to dependability, a detailed methodological description of this study was provided by the researchers.^17^ Each FGD was conducted in a private room of the unit at a convenient time agreed upon by the principal researcher and the operational manager, thus allowing for confidentiality. Before conducting the FGDs, the principal researcher and a secondary facilitator (clinical midwife) informed the participants about the purpose of the research, informed consent and the tape recording of the discussions. Participants were assured that all tape recordings would be kept confidential. The facilitators also encouraged participants to be honest, and they were assured that there were no right or wrong answers to the questions that were probed. The FGD was facilitated by the principal researcher and the secondary facilitator. All FGDs were conducted in English and within an average time of 70 minutes.

### Data analysis

Following data collection, the audiotapes were transcribed verbatim. Codes were assigned to each of the participants using the focus group number and an individual number (FGD1, P1). Braun and Clark’s^18^ approach to thematic analysis was used to analyse the transcribed data. Guest et al.^19^ describe thematic analysis as a ‘focus on identifying and describing both implicit and explicit ideas within the data, that is, themes’. Codes are then typically developed to represent the identified themes and are then applied or linked to raw data as summary markers for later analysis. The steps that were followed in the thematic analysis included (1) reading and familiarisation with the transcribed data; (2) coding; (3) searching for themes; (4) reviewing themes; (5) defining and naming themes and (6) writing up the report. Confirmability was achieved through member checking. The transcribed data were taken back to participants to review. The authors provided a ‘thick description’ of the participants and the research process.^17^ The information provided on the participants and research process would enable the reader to decide if the findings of this study could be transferable to their own setting.

### Ethical considerations

The University of KwaZulu-Natal Bioethics Research Committee (ref. no.: BFC553/16) and the KwaZulu-Natal Department of Health (ref. no.: KZ_2016RP26_545) approved this study. Permission was also granted by the Chief Executive Officer to conduct the study at the hospital facility. Written consent was obtained from all the participants.

## Results

The demographic characteristics of the participants are shown in Table 1. There were 19 female participants and one male participant, aged 25–54 years. Six overarching themes emerged from the data analysis which included perceived professional benefits of adopting the MDT care approach for adolescent mothers and their children, perceived barriers to multidisciplinary collaboration, perceived clinical benefits of adopting the multidisciplinary care approach for parenting adolescent mothers, perceived problems and needs of adolescent mothers, and nurses’ reasons regarding their willingness to participate in a multidisciplinary collaboration in the care of parenting adolescent mothers.

**TABLE 1:** Characteristics of the participants in the three focus groups.

Characteristics	Focus group 1	Focus group 2	Focus group 3	*n*	%
**Gender**					
Male	1	0	0	1	5
Female	7	6	6	19	95
**Age (years)**					
25–30	1	2	1	4	20
31–36	3	1	2	6	30
37–42	2	1	2	5	25
43–48	1	1	1	3	15
49–54	1	1	0	2	10
**Category of nursing**					
Auxiliary nurse	0	1	0	1	5
Enrolled nurse	2	1	1	4	20
Professional nurse (general stream)	2	2	3	7	35
Professional nurse (paediatrics)	0	0	2	2	10
Professional nurse (speciality in midwifery)	4	2	0	6	30
**Years of experience in the nursing profession**					
< 5 years	1	2	0	3	15
5–9 years	2	1	2	5	25
10–15 years	2	1	3	6	30
> 15 years	3	2	1	6	30

### Theme 1: Perceived professional benefits of adopting the multidisciplinary team care approach for the adolescent mothers and their children

Nurses identified the professional benefits of collaborating in an MDT to offer care to adolescent mothers and their children. These professional benefits are discussed under the following subthemes: clinical networking, improvement in communication and teamwork.

#### Clinical networking

The participants stated that clinical networking is important to allow for improved communication between healthcare providers regarding the care of the adolescent mother and her child. The absence of a clinical network to address adolescent-specific issues was clearly mentioned by participants. The quotes below reflect participant’s views on clinical networking as a benefit of adopting the MDT care approach for adolescent mothers and their children:

‘I think it’s important for us to work in a multidisciplinary team as this will improve clinical networking. As healthcare providers we can communicate to each other on ways to improve the care of parenting adolescent mothers. We can learn from each other because as healthcare providers, we are often too judgemental.’ (FGD1, P1, female, professional nurse [midwifery])

‘Currently, we don’t have a committee at the institution that focuses on pregnant and parenting adolescents. So, we really need a clinical network of healthcare providers to help each other in providing the best care to both pregnant adolescent and parenting adolescents.’ (FGD3, P3, female, enrolled nurse)

#### Improve communication

The participants believed that an MDT care approach for adolescent mothers and their children would improve communication between healthcare providers. Communication was also associated with co-ordinating the care for parenting adolescent mothers. The quotes below echo the sentiments of the participants regarding multidisciplinary collaboration and the possibility of improved communication between healthcare providers:

‘Working together as healthcare providers in a multidisciplinary team will improve our communication with regards to co-ordinating care for adolescent mothers and their children. I think it would be beneficial to the adolescent mothers if healthcare providers could address the problems of adolescent mothers and their children in a collaborative manner. So multidisciplinary teamwork can improve communication between healthcare providers. This will be very good.’ (FGD1, P6, male, professional nurse [general stream])

‘A multidisciplinary care approach will improve the clinical care of adolescent mothers and their children because the communication between nurses, social workers, psychologists and therapists will also improve.’ (FGD2, P4, female, auxiliary nurse)

#### Teamwork

Participants identified that collaborating as a team would help in finding solutions for the multitude of problems experienced by adolescent mothers and their children. The quotes below reflect participants’ views on how a team approach in healthcare delivery provides great opportunities to prioritise and assess patients’ issues and to collaborate on decisions:

‘Multidisciplinary team work will generate more efficient solutions to help the adolescent mothers and their children.’ (FGD2, P1, female, professional nurse [general stream])

‘Adolescent mothers have diverse problems so a multidisciplinary team approach will definitely help them. We as nurses cannot solve all the problems of adolescent mothers because their problems may be social or psychological. Their children may have physical and developmental problems. We need a team of specialists, e.g., social workers, psychologists, physiotherapists and speech therapists to help us to manage these problems. As a healthcare team, we can also learn from each other on how to approach problem solving.’ (FGD3, P5, female, professional nurse [general stream])

### Theme 2: Perceived potential barriers to multidisciplinary collaboration

Participants also reflected on barriers that impose on the multidisciplinary collaboration. These barriers include time constraints and shortage of staff.

#### Time constraints

Although participants acknowledged the value of multidisciplinary teamwork, some felt that they did not have adequate time for collaboration. Teamwork was also considered time-consuming especially if meetings and audits had to be conducted:

‘If we consider the many problems that children of adolescent mothers experience which include malnutrition, respiratory infections and poor personal hygiene, we need a multidisciplinary approach of care. However, I feel that we often experience time constraints that impose on us working together as a collaborative team.’ (FGD1, P3, female, enrolled nurse)

‘Even if we wanted to provide multidisciplinary healthcare services to pregnant and parenting adolescents, it is time consuming and not all healthcare providers can allocate time for meetings, clinical audits and intervention programmes. Members of the multidisciplinary team must dedicate time during their working hours which can be difficult in an already burdened system.’ (FGD2, P5, female, professional nurse [midwifery])

#### Shortage of staff

The participants highlighted that the shortage of staff prevented collaborative work as required in a multidisciplinary approach. They were concerned not only about staffing in the nursing division but also in allied health services. The following quotes illustrate the shortage of staff as a barrier to multidisciplinary teamwork:

‘We don’t have the luxury of creating a multidisciplinary team that will tailor the healthcare services to the needs of adolescent mothers because we have a shortage of nursing staff and other healthcare providers.’ (FGD1, P7, female, professional nurse [midwifery])

‘Definitely, I think a multidisciplinary care approach looking into the needs and problems of adolescent mothers is important. But insufficient staffing within the various categories of healthcare service does not allow us to implement multidisciplinary collaboration and care.’ (FGD3, P5, female, professional nurse [general stream])

### Theme 3: Perceived clinical benefits of adopting the multidisciplinary care approach for adolescent mothers and their children

The participants perceived the clinical benefits of multidisciplinary care for adolescent mothers and their children. The benefits are discussed under the following subthemes: accessibility to holistic care, increased access to tailored information, earlier diagnosis and signposting adolescent mothers and children to appropriate services, prevention of adolescent repeat pregnancies and patient satisfaction.

#### Accessibility to holistic care

Most participants felt that adolescent mothers needed both medical and psychosocial services. In this regard, participants felt that a multidisciplinary care approach would ensure that adolescent mothers and their children benefit from holistic health care:

‘I think multidisciplinary care would be beneficial to the adolescent mother because she will have access to all the services like psychology, social services, dietetics. All her problems can be addressed holistically. Currently the services are fragmented.’ (FGD1, P2, female, professional nurse [midwifery])

‘We really need holistic healthcare services and this can be achieved through multidisciplinary collaboration. I have seen a 14-year-old with repeat pregnancies and when I delved deeper, she had many issues ranging from poverty to psychological problems. It is clear that we need help from social workers, psychologists etc. While it’s important to preach the gospel of family planning and school health, we need to work as a team to deliver holistic services.’ (FGD2, P6, female, professional nurse [general stream])

#### Increased access to tailored information

The participants acknowledged that nurses might not be able to provide all the information for adolescent mothers. They also noted the importance of other specialists in providing specific information for various situations. The following quotes reflect that access to tailored information could be facilitated by teamwork:

‘Nurses are not able to provide all the information that the adolescent mother needs. Sometimes the child may have a physical problem and we have to send the child to the physiotherapist. When we work as a team, we are able to ensure the adolescent mother has access to specific information that is related to her health and the child’s health. We realise that each healthcare provider is a specialist in their field and working as a team makes it easier for the adolescent mother to get this specific information.’ (FGD2, P3, female, professional nurse [midwifery])

‘Well if we zone into the problems of adolescent mothers and their children, we will surely find that they require specific tailored solutions to their problems. We would need to establish a strong multidisciplinary team to offer tailored solutions for the problems of adolescent mothers.’ (FGD3, P3, female, enrolled nurse)

#### Earlier diagnosis and signposting adolescent mothers and children to appropriate services

The participants felt that multidisciplinary collaboration facilitates earlier diagnosis and timeous referrals to the appropriate services. They provided examples which included maternal depression and developmental problems in children:

‘The more we collaborate as a team; we appreciate the roles of other healthcare providers. Through multidisciplinary collaboration, we would also learn how to screen adolescent mothers for depression and then refer them to the psychologist.’ (FGD1, P4, female, professional nurse [general stream])

‘When you have established multidisciplinary collaboration at your institution, the communication between staff improves. We learn from each other and learn the appropriate referral procedures. During immunisation, if we detect development problems, it’s easier to refer the adolescent mothers and their children to the appropriate services if we have good teamwork.’ (FGD3, P6, female, professional nurse [paediatrics])

#### Prevention of adolescent repeat pregnancy

Adolescent repeat pregnancies were mentioned by several participants as an issue that could be prevented if healthcare providers collaborate as a team. The participants acknowledged the importance of psychosocial services (social workers and psychologists) in assisting adolescent mothers in preventing repeat pregnancies. Their opinions are illustrated in the following quotes:

‘A multidisciplinary approach to caring for adolescent mothers will help to reduce adolescent repeat pregnancy. Healthcare providers will be able to develop interventions and activities to distract adolescent mothers.’ (FGD2, P3, female, professional nurse [midwifery])

‘I think working together and using a multidisciplinary approach to care means that we improve access to social workers, psychologists and therapists. We can educate adolescent mothers on the consequences of repeat pregnancies and also prevent the repeat pregnancies.’ (FGD3, P6, female, professional nurse [paediatrics])

#### Patient satisfaction

The participants noted that adolescent mothers were often uncomfortable using healthcare services because they are grouped with adult mothers. In this regard, services were not accommodating specifically to the adolescent mother’s needs. The participants felt a multidisciplinary approach would lead to tailored services and this would improve patient satisfaction:

‘I think that a multidisciplinary approach will tailor services specifically for adolescent mothers and their children. The adolescents will be more comfortable with services that accommodate their needs. They will not be afraid to use these services.’ (FGD1, P5, female, enrolled nurse)

‘The multidisciplinary approach will surely provide a better support system for adolescent mothers and she will be more satisfied with services that deal specifically to her problems.’ (FGD3, P4, female, professional nurse [general stream])

### Theme 4: Perceived problems of adolescent mothers

The participants expressed that adolescent mothers have many problems and needs. They perceived the following as problems experienced by adolescent mothers: poor family support, poor parenting, poor attitudes of healthcare providers, poverty, peer pressure, high HIV risk, poor partner support, depression and dysfunctional parent–adolescent communication.

#### Poor family support and dysfunctional parent–adolescent communication

The participants acknowledged that most adolescent mothers experience poor family support. They stated that the adolescent pregnancies lead to anger among family members and this disrupts the family support system. Dysfunctional parent–adolescent communication was noted as a problem which worsens after the birth of the child. Adolescent pregnancies strain the relationship between parents and adolescents which leads to communication breakdown. The participants also noted that communication between the parents and the adolescent mothers may become hostile. Poor family support and dysfunctional parent–adolescent communication is illustrated by the quotes below:

‘Lack of parental support is a huge problem for the adolescent mother. Not all problems are due to the healthcare system. Attitudes of parents is a challenge. Also, discipline is lacking in most homes.’ (FGD2, P5, female, professional nurse [midwifery])

‘I think problems start at home for the adolescent mother. The parents must be approachable towards the adolescent mother and not harsh. Many girls are beaten and ill-treated by their families.’ (FGD3, P1, female, enrolled nurse)

‘Parents struggle to communicate with the pregnant adolescents. This poor relationship continues even after the baby is born. The adolescent mother is isolated. I even felt that it was difficult to communicate with my daughter when she fell pregnant at 18 years. Even after the baby was born, I felt that it was not easy to talk about the situation.’ (FGD1, P3, female, enrolled nurse)

‘Adolescent mothers have communication problems with their parents. The parents become hostile towards them due to the pregnancy and the burden of raising the child. And often parents taunt the adolescent mothers by telling them that they should have known better.’ (FGD3, P1, female, enrolled nurse)

#### Poor parenting

Adolescent parenting was considered to be of poor quality. The participants were concerned that adolescent mothers are not able to care appropriately for their children. The hospitalisation of children born to adolescent mothers was considered to be common. Recommendations for parenting education were also highlighted by participants:

‘In the paediatric ward, one will find that most of the mothers are adolescents. The children of the adolescent mothers have numerous preventable health problems. They need education and support on parenting. They often default their child’s treatment and medication.’ (FGD1, P4, female, professional nurse [general stream])

‘They don’t even know how to handle a baby. They are so scared. Maybe the occupational therapist can teach them handling and parenting skills. Demonstration with a doll will be useful to teach adolescent mothers handling skills. These mothers are babies themselves.’ (FGD3, P2, female, professional nurse [general stream])

#### Poor attitudes of healthcare providers

The participants mentioned that healthcare providers were often insolent to adolescent mothers. They felt that the stigmatisation of adolescent mothers by healthcare workers would impact the utilisation of healthcare services. Adolescent mothers were described as sensitive and healthcare workers needed to have a humane approach to caring for them. The following quotes reflect the attitudes of healthcare providers towards adolescent mothers:

‘My experiences … you find the adolescent mother arriving at the clinic late and our colleagues may become angry with her. They will shout at the adolescent mother. Their attitudes are not good and it won’t change the situation. The damage is already done. It’s stigma and attitudes of healthcare workers that pose a problem to adolescent mothers.’ (FGD1, P3, female, enrolled nurse)

‘The healthcare workers at clinics also do not support adolescent mothers. When they seek family planning services, they are mocked or scolded and don’t go back to the clinic. Bad attitudes of nurses affect the uptake of reproductive health services.’ (FGD2, P6, female, professional nurse [general stream])

#### Poverty and high HIV risk

Poverty was described as a problem for adolescent mothers and their children. The participants also noted that poverty was also leading to transactional sex and relationships with older men. The consensus was that poverty increased the vulnerabilities of the adolescent mother to further negative consequences. The participants acknowledged that the high-risk sexual behaviour of adolescent mothers makes them vulnerable to HIV infection. They also perceived that some adolescent mothers do not negotiate protective and safe sexual intercourse practices and this exposes young mothers to the HIV infection. The following quotes highlight the plight of adolescent mothers with poverty and HIV infection:

‘Poverty is a huge problem for adolescent mothers and their children. The adolescent mothers don’t have adequate financial resources for food, nappies and transport to clinics. The poverty leads to health problems for both mother and child.’ (FGD1, P7, female, professional nurse [midwifery])

‘Most of the problems affecting adolescent mothers stem from poverty. Poverty and social problems also continues to drive the cycle of adolescent repeat pregnancy. The adolescent mother also has relationships with older men for financial security because of her struggle with poverty. She may also engage in sexual activities to earn money.’ (FGD3, P4, female, professional nurse [general stream])

‘Some adolescent mothers have multiple partners or they are involved with older men. This promotes a high HIV risk because it is known that older men are more likely to infect younger women with HIV. Also having multiple partners increases their risk of HIV infection.’ (FGD2, P5, female, professional nurse [midwifery])

‘Firstly, adolescent pregnancy is a result of unprotected sex. So, this means adolescent mothers have a high risk of HIV infection. They are generally terrified of learning their HIV status. Adolescent mothers are living with HIV.’ (FGD3, P5, female, professional nurse [general stream])

#### Peer pressure

Peer pressure was described in a negative context as a factor that could lead to further problems for adolescent mothers. The participants voiced their perceptions about the problem of peer pressure in the following quotes:

‘Peer pressure is a huge problem. Adolescent mothers compete with each other. When one adolescent mother witnesses her friend receiving gifts and money from her baby daddy, then she becomes jealous. The “jealous” adolescent mother will find another partner and she will have a repeat pregnancy with a “blesser or sugar daddy”.’ (FGD1, P1, female, professional nurse [midwifery])

‘Peer pressure is evident because you find that most of the adolescent mothers are influenced by each other and have been friends in the same community.’ (FGD2, P2, female, enrolled nurse)

#### Poor partner support

Many participants voiced their concern over absent fathers. The adolescent mother was described as an isolated person who had to take sole responsibility of the child while the father is absent. The participants also felt that the paternal family needed to assist the adolescent mother and her child. The following quotes reflect the poor partner support:

‘Some adolescent mothers will be in hospital for months taking care of a sick baby but you never see the father of the child visiting or supporting her. The lack of partner support annoys me.’ (FGD2, P2, female, enrolled nurse)

‘Only the maternal family helps the adolescent mother and her child. The father of the child always disappears when the child is born. It’s time to involve the paternal family to assist the adolescent mother and her child.’ (FGD3, P1, female, enrolled nurse)

#### Depression

The participants felt that depression is a common problem among adolescent mothers which could have negative consequences for the mother and child. Loneliness and ostracisation were noted as contributing factors to adolescent maternal depression:

‘Adolescent mothers feel lonely and depression starts to develop. The depression is not good for the adolescent mother and the child. Depression can lead to child neglect.’ (FGD2, P5, female, professional nurse [midwifery])

‘Adolescent mothers may be ostracised in society and suffer depression. This affects their parenting skills.’ (FGD3, P6, female, professional nurse [paediatrics])

### Theme 5: Perceived needs of adolescent mothers

The participants perceived the following needs of adolescent mothers and their children: multidisciplinary health care, peer education, family support, parenting education, and self-efficacy and resilience.

#### Multidisciplinary health care

Multidisciplinary health care was considered as an essential need for adolescent mothers and their children. The participants reiterated that holistic health care was a combination of medical and psychosocial services:

‘Adolescent mothers need access to both medical and psychosocial problems. We need doctors, nurses, social workers, dietician[s], psychologists, therapists and even personnel from the department of basic education to work as a multidisciplinary team.’ (FGD2, P2, female, enrolled nurse)

‘A multidisciplinary approach to healthcare is a must when it involves adolescent mothers and their children because they are a vulnerable group and require specialised holistic care.’ (FGD3, P4, female, professional nurse [general stream])

#### Peer education

Peer education was considered as a valuable tool to motivate adolescent mothers and a platform to deliver sexual and reproductive health education:

‘Adolescent mothers need peer education. Bring adolescent girls who do not have children to influence adolescent mothers to take charge of their lives.’ (FGD1, P1, female, professional nurse [speciality midwifery])

‘Peer education can help break down cultural barriers and facilitate discussion on sexual and reproductive health. We need to look at peer education to prevent adolescent repeat pregnancy.’ (FGD3, P2, female, professional nurse [general stream])

#### Parenting education

The participants stated that adolescent mothers needed support on parenting. Parenting was described as a difficult task for the inexperienced adolescent mother. The following quotes identified parenting education as a need for adolescent parents:

‘Most adolescent mothers need support on parenting. It doesn’t just end with the birth of the baby. Parenting is hard and stressful. They need to know how to handle and raise their children. They don’t have experience of parenting.’ (FGD1, P7, female, professional nurse [midwifery])

‘Adolescent mothers don’t understand parenting. They often leave the children with the grandmother to raise. We need parenting education to prepare the adolescent mother to raise her child.’ (FGD3, P5, female, professional nurse [general stream])

#### Family support

Family support was described as a fundamental need of adolescent mothers. The participants felt grandmothers were an important foundation of support:

‘Families need to work with us to help adolescent mothers and their children especially grandmothers.’ (FGD1, P6, male, professional nurse [general stream])

‘We need to improve family support for adolescent mothers. We need to include parent-child family relationships and communication in the services for parenting adolescents.’ (FGD2, P3, female, professional nurse [midwifery])

#### Self-efficacy and resilience

Self-efficacy and resilience were identified as important needs for adolescent mothers. Resilience is defined as ‘the ability to successfully cope with change, misfortune or adversity’ (p. 838).^20^ Self-efficacy is defined as ‘people’s beliefs about their capabilities produce designated levels of performance that exercise over events that affect their lives’ (p. 71).^21^ The participants felt that self-efficacy would promote resilience:

‘Adolescent mothers need to educate on self-efficacy. Self-efficacy will also help improve their resilience.’ (FGD2, P6, female, professional nurse [general stream])

‘Adolescent mothers need self-love, confidence and improved self-esteem. Most importantly they need to be resilient.’ (FGD3, P1, female, enrolled nurse)

### Theme 6: Nurses’ reasons for their willingness to participate in a multidisciplinary team for the care of adolescent mothers and their children

The reasons that participants cited with regard to their willingness to participate in an MDT for the care of adolescent mothers and their children included improvement in service delivery, personal interests, magnitude of the problem and knowledge exchange.

#### Improvement in service delivery

The participants were willing to engage in multidisciplinary teamwork because they felt that this approach would improve the service delivery to adolescent mothers and their children. The families of the adolescent mothers were also considered as team members in the multidisciplinary approach. The following quotes illustrate that participants’ willingness to take part in multidisciplinary teamwork is influenced by the potential to improve service delivery:

‘I believe that having a multidisciplinary approach will improve the clinical services for adolescent mothers and their children. Teamwork will generate more efficient solutions and we need holistic care such as working with families to help the adolescent parents.’ (FGD1, P1, female, professional nurse [midwifery])

‘If we have multidisciplinary teams to deal specifically with adolescent pregnancy and parenting, we will establish strong clinical services and also improve overall service delivery.’ (FGD3, P6, female, professional nurse [paediatrics])

#### Personal interests

Prior experience with adolescent pregnancy provided a personal motivation to some participants who want to engage in multidisciplinary teamwork. Empathy was also strongly emphasised as a willingness to actively participate in caring for adolescent mothers and their children. Participants reflected on how their personal experiences have shaped their interest in participating in a multidisciplinary care approach for pregnant and parenting adolescent mothers in the following quotes:

‘I am on board when it involves multidisciplinary care because I am a mother. It hurts me to see young women struggling in life financially while trying to raise their children. I also see many malnourished and abused children whose parents are adolescents.’ (FGD2, P5, female, professional nurse [midwifery])

‘I was an adolescent mother and I wish that there would have been more support from healthcare providers. I was fortunate that my grandmother assisted me in caring for my child. I have empathy for adolescent mothers. I would strongly advocate for multidisciplinary care of adolescent mothers.’ (FGD3, P3, female, enrolled nurse)

#### Magnitude of the problem

The participants emphasised the magnitude of the problem of adolescent pregnancy in the district. In this regard, they felt a sense of responsibility to actively participate in an MDT to resolve issues around adolescent pregnancy and motherhood. The magnitude of adolescent pregnancy and motherhood is echoed in the following quotes:

‘I would participate in multidisciplinary teams to help adolescent mothers because there are more adolescent mothers delivering at this institution.’ (FGD1, P4, female, professional nurse [general stream])

‘Adolescent pregnancy is a huge and common problem in this district. It is also a vicious cycle. I would participate in multidisciplinary teamwork to help decrease the repeat adolescent pregnancies which is also a big problem.’ (FGD2, P1, female, professional nurse [general stream])

#### Knowledge exchange

The participants felt that with the multidisciplinary approach they would benefit from learning and sharing solutions regarding the care of pregnant and parenting adolescents. The knowledge exchanges would motivate participants to actively engage in multidisciplinary work:

‘I would participate actively in a multidisciplinary team caring for adolescent mothers because we could learn from each other. After all knowledge is power.’ (FGD2, P3, female, professional nurse [midwifery])

‘I think that we learn from each other about the consequences of early childbearing. The sharing of knowledge can help us build around the current services to help pregnant and parenting adolescents. I am keen to learn from my colleagues.’ (FGD3, P4, female, professional nurse [general stream])

## Discussion

The findings of our study indicate that nurses perceive a multidisciplinary approach that addresses the medical and psychosocial concerns which is important in the care of pregnant and parenting adolescent females. MDT s provide benefits to both service users and healthcare providers. Spruyt^22^ found that MDTs develop clinical networking between healthcare providers. Clinical networking supports information sharing between health professionals. Effective multidisciplinary teamwork improves communication between members of the team. Teamwork also fosters partnership, friendship and support especially in difficult clinical circumstances.^22,23^

Multidisciplinary teamwork is also plagued by barriers. The common barriers identified in the literature include time constraints and shortage of staff.^24^ Similarly, the participants in our study also emphasised time constraints and shortage of staff. Healthcare providers find that there is insufficient time outside clinical work that can be devoted to multidisciplinary teamwork.^24^ Teamwork is also viewed as time-consuming. A study conducted in the United Kingdom found that nurses perceived staff shortages as a barrier that hindered multidisciplinary teamwork.^25^ Although, there is limited information on multidisciplinary care of adolescent mothers and their children in South Africa, we draw information from other studies on the subject of multidisciplinary teamwork. In the South African context, the multidisciplinary rehabilitation of patients with disabilities was hindered by staff shortages.^26^

Multidisciplinary collaboration provides an opportunity for good quality holistic health care.^23,24,27^ The sexual and reproductive health needs of adolescent mothers include access to contraception, treatment of sexually transmitted infections (STI) and prevention of repeat pregnancy.^27^ Adolescent mothers are also in need of psychosocial services to address interpersonal violence, abuse, trauma, unemployment, poverty and depression.^27^ Research carried out in the CARE clinic, Singapore, for pregnant adolescents found that access to antenatal, postnatal and psychosocial services improved through a multidisciplinary approach.^26^ Sexually transmitted infection screening services in the CARE clinic increased from 50% in 2008 to 85.3% in 2012. Contraceptive uptake by adolescent mothers postpartum also increased. Swallow et al.^28^ found that healthcare providers in a hospital-based MDT acknowledged the benefits of tailored clinical information regarding patient care. The Canadian Paediatric and Adolescent Gynaecology and Obstetrician Committee (CANPAGO) recommend that healthcare providers adapt their services to the needs of pregnant adolescents.^29^ Licitra et al.^30^ note that MDT discussions assist in the facilitation of earlier diagnosis and timeous referral of patients to the appropriate services. Adolescent pregnancy is considered a high-risk pregnancy whereby the earlier diagnosis of complications and timeous referral is important for maternal and child health outcomes.^8^

Repeat pregnancies among adolescent mothers are common.^31^ The participants in this study reiterated the need to prevent adolescent repeat pregnancies. An adolescent with multiple pregnancies during her adolescent phase is at a higher risk of medical, psychological and social complications than older mothers.^32^ A subsequent rapid repeat pregnancy further delays an adolescent’s chances of completing her education and seeking employment.^33^ Repeat adolescent pregnancies intensifies the cycle of poverty. Adolescent mothers receiving MDT interventions comprising medical, educational, psychosocial and family planning had lower incidences of repeat pregnancies than adolescents who received conventional care.^34^ Although the literature is limited regarding adolescent mothers’ satisfaction with the multidisciplinary approach of care, several studies within the healthcare context report that patients who received care from an MDT were satisfied with the care.^35,36^

Adolescent pregnancy does not occur in a vacuum. The effects of adolescent childbearing are experienced by the family members. The pregnancy and birth of the child may create family conflict and result in poor family support for the adolescent mother.^37^ The adolescent mother may experience a struggle between feelings of joy, abandonment and despair. In this regard, psychosocial support is required to assist families and the adolescent mother. Apart from family conflicts, adolescent mothers may experience problems with parenting. Factors that are known to affect parenting include intergenerational factors, family support, partner support and stress.^38^ Young adolescent mothers may be egocentric and prioritise their needs prior to that of their infants.^39^ They may develop feelings of resentment if they find parenting too demanding and a hindrance to their social lives.^40^ Adolescent parenting can have negative consequences for the mother, father, child and the grandparents.^41^

Early adolescent pregnancy in the Eastern Cape, South Africa, was found to be associated with a higher risk of HIV infection.^42^ The risk factors attributed to HIV infection among adolescent females included risky sexual behaviour and relationships with older partners. Similarly, Horwood et al.^43^ noted a high HIV prevalence among adolescent mothers in KwaZulu-Natal, South Africa. The participants in our study also argued that the risk of HIV infection among adolescent mothers is high. A multidisciplinary approach which includes adolescent-friendly family planning services and prevention of mother-to-child treatment (PMTCT) services is essential. Adolescent mothers’ uptake of reproductive health services is also hampered by the attitudes of healthcare providers.^44^ A questionnaire survey among nurse midwives in Kenya and Zimbabwe revealed that nurse midwives in both countries condemned adolescent sexual activity, masturbation, abortion and contraception use.^45^ Similarly, an adolescent reproductive and sexual health study in Thailand revealed that sexually active adolescents preferred to use private pharmacies for self-medication, and they would only seek health care at a government health facility as a last option because of the lack of privacy and the judgemental attitudes of healthcare workers towards sexually active adolescents.^44^

### Limitations of the study

This study was conducted in one district hospital in a rural area, and the findings of the study therefore can only be transferred to a similar research setting. The number of participants was limited and therefore the focus groups were a few.

## Conclusion

It is noteworthy that the common needs identified for parenting adolescent mothers include multidisciplinary health care, peer education, family support, adolescent parenting education, self-efficacy and resilience. Nurses’ willingness to participate in a multidisciplinary care approach was influenced by potential improvement in service delivery, personal interests, and the magnitude of the problem and knowledge exchange. Family support and psycho-education enhance resilience and positive parenting behaviour of adolescent mothers.^45^ Self-efficacy beliefs influence resilience. Firth-Cozens^26^ emphasises that:

[…] teams make up the building blocks of health care and every team from the executive to the coal-face is composed of different professionals, ideally possessing a variety of skills necessary to produce safe and effective care. (p. 65)

In the case of nurses, the MDT approach of care for adolescent mothers and their children is an important strategy to improve maternal and child health outcomes. The findings of this study have important implications for the development of an intervention.

## References

[1] Skobi F, Makofane M Reflections of social workers on the experiences of pregnant teenagers during group work sessions. Social Work. 2017;53(5):242–249. 10.15270/52-2-566/socialwork.journals.ac.za

[2] United Nations Population Fund Adolescent pregnancy: A Review of the Evidence [homepage on the Internet]. New York: United Nations Population Fund 2013 [cited 2016 Apr 20]. Available from: https://www.unfpa.org/sites/default/…/ADOLESCENT%20PREGNANCY_UNFPA.pdf

[3] Queiroz MVO, Menezes GMD, Silva TJP, Brasil EGM, Silva RM Pregnant teenagers’ group: Contributions to prenatal care. Rev Gaúcha Enferm. 2016;37:e20160029 10.1590/1983-1447.2016.esp.2016-0029 28640332

[4] Jutte D, Roos N, Brownell M, Briggs G, MacWilliam L, Roos LL The ripples of adolescent motherhood: Social, educational and medical outcomes for children of teen and prior teen mothers. Acad Pediatr. 2010;10(5):293–301. 10.1016/j.acap.2010.06.008 20674531

[5] Keown L, Woodward LJ, Field J Language development of pre-school children born to teenage mothers. Infant Child Dev. 2001;10:129–145. 10.1002/icd.282

[6] Clincy AR, Mills-Koonce WR Trajectories of intrusive parenting during infancyand toddlerhood as predictorsof rural, low-income African American boy’s school-related outcomes. Am J Orthopyschiatry. 2013;83(203):194–206. 10.1111/ajop.12028 PMC4439403 23889012

[7] Thompson G Meeting the needs of adolescent parents and their children. Paediatrics & Child Health. 2016;21(5):273. 27441025 10.1093/pch/21.5.273 PMC4933062

[8] Benagiano G, Brosens I The multidisciplinary approach. Best Pract Res Clin Obstetrics Gynaecol. 2014;28:1114e–1122e. 10.1016/j.bpobgyn.2014.08.006 25199857

[9] Mitchell G, Tieman J, Shelby-James T Multidisciplinary care planning and teamwork in primary care. MJA. 2008;188:s61–s64. 18429739 10.5694/j.1326-5377.2008.tb01747.x

[10] Pinzon JL, Jones VF Care of adolescent parents and their children. Paediatrics. 2012;130(6):1743–1756. 10.1542/peds.2012-2879 23184113

[11] Quinlivan JA, Box H, Evans S Postnatal home visits in teenage mothers: A randomised controlled trial. Lancet. 2003;361:893–900. 10.1016/S0140-6736(03)12770-5 12648967

[12] Govender T, Reddy P, Ghuman S Obstetric outcomes and antenatal access among adolescent pregnancies in KwaZulu-Natal, South Africa. S Afr Fam Pract. 2017;60:1–7. 10.1080/20786190.2017.1333783

[13] Sodi E, Sodi T Quality of daily life with teenage motherhood. J Psychol Afr. 2012;22(3):429–434. 10.1080/14330237.2012.10820550

[14] Koshy E, Koshy V, Waterman H Action Research in Healthcare. Thousand Oaks, CA: Sage; 2011.

[15] Reason P, Bradbury H Introduction In Reason P, Bradbury H, editors The Sage handbook of action research: Participative inquiry and practice. Thousand Oaks, CA: Sage, 2008; p. 1–9.

[16] Moonsamy P GJ Crookes Hospital Informatics Report. Ugu: GJ Crookes Hospital, KwaZulu Natal Department of Health; 2018.

[17] Shenton AK Strategies for ensuring trustworthiness in qualitative research projects. Educ Inform. 2004;22:63–67. 10.3233/EFI-2004-22201

[18] Braun V, Clark V Using thematic analysis in psychology. Qual Res Psychol. 2006;3(2):77–101. 10.1191/1478088706qp063oa

[19] Guest G, MacQueen KM, Namey EE Applied thematic analysis. Thousand Oaks, CA: Sage; 2011.

[20] Sagone E, Elvira De Caroli M Relationships between psychological well-being and resilience in middle and late adolescents. Procedia-Social Behav Sci. 2014;141:881–887. 10.1016/j.sbspro.2014.05.154

[21] Bandura A Self-efficacy In Ramachaudran VS, editor Encyclopedia of human behavior. San Diego, CA: Academic Press, 1994; p. 71–81.

[22] Spruyt O Team networking in palliative care. Indian J Palliat Care. 2011;17:S17–S19. 10.4103/0973-1075.76234 21811361 PMC3140085

[23] Firth-Cozens J Multidisciplinary teamwork: The good, bad, and everything in between. Qual Health Care. 2001;10:65–66. 10.1136/qhc.10.2.65 11389310 PMC1757983

[24] Carter S, Garside P, Black A Multidisciplinary team working, clinical networks, and chambers; opportunities to work differently in the NHS. Qual Safe Health Care. 2003;12:i25–i28. 10.1136/qhc.12.suppl_1.i25 PMC1765763 14645745

[25] Atwal A, Tattersall K, Caldwell K, Craik C Multidisciplinary perceptions of the role of nurses and healthcare assistants in rehabilitation of older adults in acute health care. J Clin Nurs. 2006;15:1418–1425. 10.1111/j.1365-2702.2005.01451.x 17038103

[26] Ennion L, Rhoda A Roles and challenges of the multidisciplinary team involved in prosthetic rehabilitation, in a rural district in South Africa. J Multidiscip Healthc. 2016;9:565–573. 10.2147/JMDH.S116340 27826195 PMC5096738

[27] Kwek LK, Sulaiman S, Hassan Y, Nadarajah S Clinic for the pregnant adolescent (CARE) – 5 years on. Obstet Gynecol Int J. 2016;5(5):00172 10.15406/ogij.2016.05.00172

[28] Swallow V, Smith T, Webb NJA, et al Distributed expertise: Qualitative study of a British network of multidisciplinary teams supporting parents of children with chronic kidney disease. Child Care Health Dev. 2014;41:67–75. 10.1111/cch.12141 24827413 PMC4368419

[29] Fleming N, O’Driscoll T, Becker G, Calgary AB, Spitzer RF Adolescent pregnancy guidelines. J Obstet Gynaecol Canada. 2015;37(8):740–756. 10.1016/S1701-2163(15)30180-8 26474231

[30] Licitra L, Keiholz U, Tahara M, et al Evaluation of the benefit and use of multidisciplinary teams in the treatment of head and neck cancer. Oral Oncol. 2016;59:73–79. 10.1016/j.oraloncology.2016.06.002 27424185

[31] Viera CL, Flores PV, de Carmargo KR, et al Rapid repeat pregnancy in Brazilian adolescents: Interaction between maternal schooling and age. J Paediatr Adolesc Gynaecol. 2016;29:382–385. 10.1016/j.jpag.2016.01.121 26860545

[32] Raneri LG, Weimann M Social ecological predictors of repeat adolescent pregnancy. Perspect Sex Reprod Health. 2007;39(1):39–47. 10.1363/3903907 17355380

[33] Lewis LN, Doherty DA, Hickey M, Skinner SR Predictors of sexual intercourse and rapid repeat pregnancy among teenage mothers: An Australian prospective longitudinal study. Med J Aust. 2010;193(6):338–342. 20854238 10.5694/j.1326-5377.2010.tb03944.x

[34] Govender D, Naidoo S, Taylor M Scoping review of risk factors and interventions for adolescent repeat pregnancies: A public health perspective. Afr J Prim Health Care Fam Med. 2018;10(1):a1685 10.4102/phcfm.v10i1.1685 PMC6018382 29943611

[35] Ngubane M, Chetty V Caregiver satisfaction with a multidisciplinary community-based rehabilitation programme for children with cerebral palsy in South Africa. S Afr Fam Pract. 2017;59(1):35–40. 10.1080/20786190.2016.1254929

[36] Janssen RMJ, Satink T, Ijspeert J, et al Reflections of patients and the multidisciplinary rehabilitation programme for persons with brachial plexus injuries. Disabil Rehabil. 2018;40 10.1080/09638288.2018.1430175 29385821

[37] Maranha TA, Gomes KRO, De Oliveira DC Couple and family relationships post pregnancy. Acta Paul Enferm. 2012;25(3):371–377.

[38] Easterbrooks MA, Chaudhuri JH, Bartlett JD, Copeman A Resilience in parenting among young mothers: Family and ecological risks and opportunities. Child Youth Serv Rev. 2011;33:42–50. 10.1016/j.childyouth.2010.08.010

[39] Leslie K, Dibden L Adolescent parents and their children: The paediatrician’s role. Paediatr Child Health. 2004;9(8):561–564. 10.1093/pch/9.8.561 19680485 PMC2724164

[40] Ward C, Makusha T, Bray R Parenting, poverty and young people in South Africa: What are the connections? In De Lanay A, Swart S, Lake L, Smith S, editors, South African Child Gauge. Cape Town: South African Children’s Institute: University of Cape Town, 2015; p. 69–74.

[41] Christofides NJ, Jewkes RK, Dunkle KL, Nduna M, Shai NJ, Sterk C Early adolescent pregnancy increases risk of incident HIV infection in the Easter Cape, South Africa. J Int AIDS Soc [serial online]. 2014;17:e18585 [cited 2018 Aug 22]. Available from: http://www.jiasociety.org/index.php/jias/article/view/18585 10.7448/IAS.17.1.18585 PMC3962027 24650763

[42] Horwood C, Butler LM, Haskins L, Phakathi S, Rollins N Adolescent mothers and their infants: Low coverage of HIV services and high risk of HIV transmission in KwaZulu-Natal. PLoS One. 2013;8(9):e74568 https://doi:10.1371/journal.pone.0074568 24073215 10.1371/journal.pone.0074568 PMC3779214

[43] Warenius LU, Faxelid EA, Chishimba PN, Musandu JO, Ong’any AA, Nissen EBM Nurse-Midwives’ attitudes towards adolescent sexual and reproductive health needs in Kenya and Zambia. Reprod Health Matters. 2006;14(27):119–128. 10.1016/S0968-8080(06)27242-2 16713886

[44] Tangmunkongvorakul A, Banwell C, Carmichael G, et al Use and perceptions of sexual and reproductive health services among young northern Thai people. Southeast Asian J Trop Med Public Health. 2012;43(2):479–500. 23082599 PMC3732782

[45] McGaha-Garnett V Needs assessment for adolescent mothers: Building resiliency and student success towards high school completion In Walz R, Bleuer JC, Yep RK, editors Compelling counselling interventions. Ann Arbor, MI: Counselling Outfitters; American Counselling Association, 2008; p. 11–20.

